# The effects of sexual violence on psychosocial outcomes in formerly abducted girls in Northern Uganda: the WAYS study

**DOI:** 10.1186/s40359-015-0103-2

**Published:** 2015-12-22

**Authors:** Kennedy Amone-P’Olak, Emilio Ovuga, Peter Brian Jones

**Affiliations:** Department of Psychology, University of Botswana, Private Bag UB 00705, Gaborone, Botswana; Department of Psychiatry and Mental Health, Gulu University, P O Box 166, Gulu, Uganda; Department of Psychiatry, Herchel Smith Building for Brain & Mind Sciences, Forvie Site, Robinson Way, Cambridge, CB2 0SZ UK

## Abstract

**Background:**

The objective of this study is to investigate the effects of sexual violence on the odds of different psychosocial outcomes (depression, psychotic symptoms, somatic complaints, conduct problems, daily functioning, community relations, and stigma) among formerly abducted girls in Uganda.

**Methods:**

Data from an on-going **W**ar-**A**ffected **Y**outh **S**tudy (WAYS) in Uganda was used to compute the prevalence of psychosocial problems (scores ≥ 75th percentile) among three categories of formerly abducted girls (1) no history of sexual violence without children, 2) a history of sexual violence without children, and 3) a history of sexual violence with children as a consequence) among 210 women (age 22.06, SD = 2.06, range 18–25). Multiple logistic regression analyses were used to examine differences in psychosocial outcomes by the different categories of formerly abducted girls.

**Results:**

Compared to participants with no history of sexual violence and without any children, the odds of adverse psychosocial outcomes were increasingly higher for all psychosocial dimensions for those who reported sexual violence with or without children. Those with a history of sexual violence and with children as a consequence had more than five times the odds of reporting depressive symptoms (OR, 5.37; 95 % CI (1.45–19.90), somatic complaints (OR, 6.59; 95 % CI (1.80 – 24.11), and stigma (OR, 13.85; 95 % CI (3.73 – 51.42) compared to those who did not report sexual violence.

**Conclusion:**

This study highlighted the risks of psychosocial problems among different categories of formerly abducted girls regarding sexual violence. Vulnerability to psychosocial problems among formerly abducted girls is further compounded by sexual violence, child care, stigma, and poverty.

## Background

Considerable evidence has accumulated linking sexual violence during war to long-term physical, psychological and social problems [1–5]. Physical problems include genital injury and fistulae, while psychological ones include posttraumatic stress disorder (PTSD) and depression, and the social problems comprise stigma and poor community relations [6]. In the Democratic Republic of the Congo (DRC), Sierra Leone, and Northern Uganda, war-time sexual violence has been systematic, widespread, and perpetrated with impunity against young girls and women over a long period of time [1–3, 7]. In Northern Uganda, girls and young women were abducted, taken into rebel captivity and forced into sexual servitude, often distributed as “wives” to loyal rebel soldiers and commanders and playing other roles such as combat, caring for the wounded and sick, performing domestic work, and working as maids and minders of the children of senior rebel commanders. Many of the girls returned from captivity with children fathered by rebel soldiers and commanders [7–9].

In spite of the endemic sexual violence in these war-torn countries, few systematic studies have been carried out to assess the psychosocial effects on survivors in the aftermath of the wars [10, 11]. Previous studies focused mainly on documenting the incidents of sexual violence on young girls and women in Liberia [12, 13], Sierra Leone [6], DR Congo [2], and Northern Uganda [7, 14]. Similarly, no studies have specifically quantified the psychosocial impact of sexual violence on survivors in low resource settings with a view to inform interventions, policy, and research. Moreover, most previous studies regarded survivors of sexual violence as a homogenous group without considering the possibility that there could be different categories of survivors such as those with or without children born as a result of sexual violence. Understanding that survivors of sexual violence are not homogenous is critical to planning treatment, reintegration, and designing interventions.

Coupled with the negative traditional view of having children out of wedlock or as result of rape, survivors of sexual violence as a result of the war in Northern Uganda face enormous psychosocial problems [15, 16]. For example, raising children born in rebel captivity as a result of sexual violence is likely to be associated with additional burden of care and psychological distress for survivors, thus limiting opportunities in life such as continuing with education, training for a skill, or even getting married.

Using data from an on-going longitudinal study in Northern Uganda, we sought to investigate the impact of sexual violence on psychosocial outcomes (depression/anxiety, somatic complaints, conduct problems, stigma, community relations, and daily functioning) among formerly abducted girls in Northern Uganda. Specifically, we aimed to: (a) determine whether demographic characteristics such as age at abduction, age during baseline, and duration in captivity were related to psychosocial outcomes, (b) determine the prevalence of psychosocial problems among survivors of sexual violence during the war in Northern Uganda, and (c) examine the differential effects of sexual violence on a variety of psychosocial dimensions for three categories of formerly abducted girls with: 1) no history of sexual violence, 2) a history of sexual violence but with no children as a consequence, and 3) a history of sexual violence with children as a consequence. We hypothesised that the risks of adverse psychosocial outcomes in formerly abducted girls and young women increases with sexual violence and having a child or children as a consequence.

## Methods

The **W**ar-**A**ffected **Y**outh **S**urvey (the WAYS study) is a large longitudinal survey that aims to chart and illuminate the course of post-war psychosocial outcomes in the context of individual, family and community factors in war-affected youths in Northern Uganda. Northern Uganda endured a two-decade war (1986–2006) in which thousands of children including girls and young women were abducted and taken into captivity where they were forced into sexual servitude. The cohort profiles, details of the methods, and descriptions of the outcomes are reported elsewhere [17].

### Participants

The WAYS study recruited participants from five districts of Northern Uganda (Gulu, Nwoya, Amuru, Pader, and Kitgum) severely affected by the twenty-year war using a multi-stage cluster sampling strategy. Initially, lists of formerly abducted children drawn by UNICEF with the help of local governments and the community were obtained. Subsequently, participants were eligible to participate on the basis of the following criteria: 1) a history of abduction by rebels, (2) having lived in rebel captivity for at least 6 months, and (3) aged between 18 and 25 years. Next, local leaders invited those who met the inclusion criteria to participate in the study. The baseline survey was carried out from June to September 2011.

### Data collection

University graduates were recruited and thoroughly trained as research assistants to collect data for the study. Data across a variety of domains (e.g. demographic characteristics, sexual violence, and common symptoms of depression, anxiety, conduct problems, somatic complaints, psychotic symptoms, stigma, community relations, and daily functioning) were collected using questionnaires from participants’ villages or nearby trading or community centres. Prior to the start of the study, the questionnaires were pilot tested to establish reliability, validity, and feasibility. Participants spent between 30 – 45 min to complete the questionnaire.

### Ethical considerations

The WAYS study was approved by the Institutional Review Board (IRB) of Gulu University, an affiliate of Uganda National Council for Science and Technology (UNCST), the overall body that oversees research in Uganda. Participants gave written informed consent in accordance with ethical guidelines and approvals. All participants received a T-shirt each after the interview sessions in appreciation for their time and participation. No other incentives were given. A Clinical Psychiatric Officer was always on site to make referrals to the Regional Referral Hospital in case of mental health emergency such as severe depression, suicidal behaviour, homicide, or conduct problem with a potential for harm.

### Measures

The measures used for the current study were back-translated from English to Luo, the native language of the participants, by experts who are fluent in both the English language and Luo.

#### Demographic characteristics

An inventory specifically designed for this study was used to elicit information on sex, age at abduction, age at baseline, duration in captivity, and children born while in captivity.

#### Sexual violence

Sexual violence was elicited using a single-item question from the UNICEF B&H (Bosnia Herzegovina) Post-war Screening Survey [18]. The item inquired whether participants were sexually abused during abduction or in rebel captivity or not. The response was binary coded as “1” for occurrence and “0” for absence of sexual abuse.

#### Mental health outcomes (depression/anxiety, conduct problems, somatic complaints, and psychotic symptoms)

The African Youth Psychosocial Assessment Instrument (APAI), a field-based measure previously developed for use in Northern Uganda was used to elicit mental health outcomes (depression/anxiety, conduct problems, and somatic complaints) [19, 20]. The measure comprises items on depression/anxiety [18 items], conduct problems [ten items], and somatic complaints without medical cause [three items]). Each of these dimensions is represented by a set of questions that inquires about specific behaviours particular to that dimension (e.g. “I do not sleep at night” [Depression], “I fight” [conduct problems], and “I have pain all over my body” [Somatic complaints]. The responses were scored on a four-point Likert scale ranging from 0–3 scale with 0 = never, 1 = rarely, 2 = sometimes, and 3 = always with a higher score indicating that a participant would have more symptoms of a particular mental health outcome (e.g. depression/anxiety).

In the current study, psychotic symptoms (i.e., hallucinations, delusions, and persecutory feelings) were not part of APAI and were assessed using the following four items: (1) sometimes I hear voices or see things other people do not see, (2) sometimes I feel that I have special powers, (3) sometimes I think that people are listening to my thoughts or watching me when I am alone, and (4) sometimes I think that people are against me. The four items covered hallucinations, delusions, and persecutory feelings, all common features of psychotic symptoms. The items were scored on a four-point Likert scale: 0 = never, 1 = rarely, 2 = sometimes, and 3 = always. The psychotic symptoms scale had good psychometric properties (Cronbach’s alpha = .71).

#### General functioning

General functioning was indicated by difficulties performing daily tasks and activities. This measure was derived by earlier qualitative study of the experiences of war-affected youths in Sierra Leone and Uganda [10, 19, 20]. A 13-item questionnaire rated on a four-point Likert scale from 1 = not difficult to 4 = very difficult was used to assess general functioning. This scale included items assessing levels of difficulties participating in the following activities: fetching water or firewood, participation in social functions such as traditional dances, community gatherings such as funerals or marriage ceremonies, domestic hygiene, etc. General functioning was indicated by the sum scores ranging from 1 to 52 with higher scores indicating poor functioning. The Cronbach alpha for this scale was 0.84 for the current study.

#### Stigma

Stigma was assessed by a 9-item Everyday Discrimination Scale [21]. The questionnaire inquires about the extent to which they agree to statements where most people undervalue formerly abducted children, regard them as failures and less intelligent than others, and as individuals whose opinions need not be taken seriously. The questionnaire included items such as: “sometime I feel I am being talked down to because of having been in rebel captivity” and “people have insulted me because of having been in rebel captivity”. The response format was based on a five-point Likert scale with 1 = strongly disagree and 5 = strongly agree with higher scores indicating greater perception of stigma. The Cronbach alpha reliability for this scale was .87.

#### Community relations

Perceptions of common expressions of approval or recognition from others in their community was assessed with a six-item questionnaire. This measure was derived by earlier qualitative study of the experiences of war-affected youths in Sierra Leone and Uganda [10, 19, 20]. The measure included items such as “since the war, people in this community have been good to you” and ‘since the war, you feel you have been welcomed back into the community where you live.” Unlike items on the stigma scale, those on the community relations’ scale were not worded to particularly refer to the experience of having been a former child soldier. The items on this scale were scored on a three-point Likert scale with response options of 0 = “not true” to 1 = “sometimes true” or 2 = “very true” with higher scores indicating poor community relations. The Cronbach’s alpha for this scale in this study was *α* = .87.

We dichotomized the psychosocial outcomes such that scores above 75th percentile were arbitrarily selected to identify an impaired group for two reasons: First, information on an impaired group is important for identifying those at risk of psychosocial problems and for targeting intervention, thus making the possible clinical implications of our study of greater public health relevance. Second, preliminary analyses had indicated that there were significant differences between those with adverse scores (≥75th percentile) and those with “normal” (scores less than the 75th percentile) scores on psychosocial outcomes with regard to daily functioning. In a previous article from the same research project, we dichotomised at ≥ 75th percentile to demarcate the presence or absence of difficulty with general functioning (Amone-P’Olak, Jones, Meiser-Stedman, et al. 2014) [15].

### Statistical analyses

Even though the WAYS study is a longitudinal cohort study, the design for our analysis was cross-sectional. First we assessed the potential of age at abduction, duration in captivity, and age at baseline to confound the association between sexual violence and psychosocial outcomes. We grouped participants into three categories of sexual violence in order to allow for comparisons:

1. No history of sexual violence and without any children;
2. A history of sexual violence without any children;
3. A history of sexual violence with children as a consequence.

To examine whether sexual violence was associated with poor psychosocial outcomes, the prevalence of dimensions of psychosocial outcomes (depression/anxiety, psychotic symptoms, conduct problems, somatic complaints, stigma, community relations, and general functioning), was computed on the basis of whether participants experienced sexual violence or not and whether they had children as a consequence. We used binary logistic regression analyses to quantify the associations between different exposures to sexual violence and psychosocial outcomes to obtain odds ratios and 95 % Confidence Intervals (95 % CI). In these analyses the presence of each separate psychosocial outcome (defined as ≤ 75th percentile) was the dependent variable and categories of exposure to sexual violence (i.e. no history of sexual violence and no children, a history of sexual violence but with no children as a consequence, and a history of sexual violence with children as a consequence) were entered as categorical independent variables with those with a history of sexual violence and no child used as a reference category. Considered a potential confounder, age at abduction, duration in captivity, and age at baseline were additionally entered into the binary logistic regression models. To ensure all variables in the mediation models were comparable, we standardized them to a mean of zero and SD of 1 (Z scores). All analyses were conducted using Stata statistical software (version 13): release 2013 [22]. Clustering by district and non-response were accounted for using relevant survey commands in Stata.

## Results

### Demographic characteristics and prevalence of sexual violence

In this study, we analysed data from 210 formerly abducted girls (mean age = 22.39, SD = 2.03, age range = 18–25). The demographic characteristics of and bivariate correlations between variables in the study are described and presented in Table 1. Specifically, the psychosocial (stigma, community relations, and general functioning) and mental health (depression/anxiety, conduct problems, psychotic symptoms, and somatic complaints) outcomes were moderately correlated with each other. In general, there were weak and often non-significant associations between demographic characteristics (e.g. age at abduction, duration in captivity, and age at baseline) and psychosocial outcomes (e.g. depression/anxiety, somatic complaints, stigma/discrimination, and daily functioning). Age at abduction correlated significantly with only stigma/discrimination while daily functioning correlated negatively with duration in captivity (Table 1).

**Table 1:** Bivariate correlations between continuous measures of demographic characteristics and different psychosocial outcomes and their mean values

		Mean	SD	Range	1	2	3	4	5	6	7	8	9	10
1	Age at abduction	11.58	11.30	08–20	1									
2	Duration in captivity	3.48	3.40	0.5–15	−0.01	1								
3	Age at baseline	22.06	2.01	18–25	**0.14** ^a^	0.11	1							
4	Depression/anxiety	24.71	10.54	04–50	0.12	−0.06	0.01	1						
5	Psychotic symptoms	4.18	2.52	00–12	0.05	−0.06	−0.02	**0.46** ^b^	1					
6	Conduct problems	2.26	3.19	00–19	0.07	−**0.20** ^b^	0.04	**0.31** ^b^	**0.23** ^b^	1				
7	Somatic complaints	4.49	2.14	00–09	0.06	−0.01	0.11	**0.58** ^b^	**0.37** ^b^	**0.27** ^b^	1			
8	Stigma/discrimination	39.94	9.37	13–58	**0.15** ^a^	−0.04	0.01	**0.45** ^b^	**0.47** ^b^	**0.25** ^b^	**0.32** ^b^	1		
9	Community relations	8.17	4.00	00–18	0.02	−0.08	0.02	**0.34** ^b^	**0.45** ^b^	0.09	**0.33** ^b^	**0.62** ^b^	1	
10	Daily functioning	16.66	11.25	00–24	0.02	**−0.17** ^a^	0.08	**0.17** ^a^	**0.24** ^b^	0.01	0.11	**0.28** ^b^	**0.42** ^b^	1

Significant correlations are in bold figures

^a^Correlation is significant at the 0.05 level (2-tailed)

^b^Correlation is significant at the 0.01 level (2-tailed)

Of the 210 participants, 135 (65 %) reported sexual violence while in rebel captivity and 50 % (*n* = 67) returned with at least a child or children fathered by rebel commanders or soldiers.

### Prevalence of psychosocial problems in formerly abducted girls

**Fig. 1:**
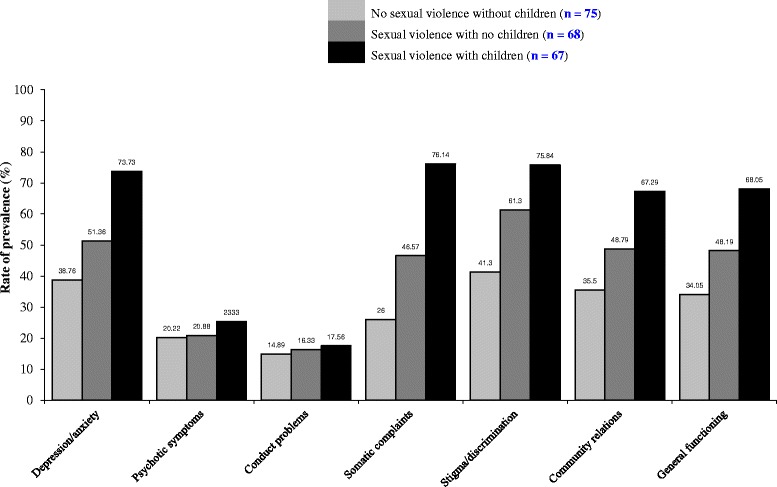
Prevalence of psychosocial problems among formerly abducted girls in Northern Uganda

The prevalence of psychosocial problems according to different experiences of sexual violence with and without children is presented in Fig. 1. History of abduction without sexual violence, experience of sexual violence without any children, and experience of sexual violence with children as a consequence, was gradually associated with a higher prevalence of all psychosocial problems. The gradients were steeper for all psychosocial outcomes except psychotic symptoms and conduct problems (Fig. 1). Except for psychotic symptoms and conduct problems, the prevalence (indicated by ≥ 75th percentile) of the other psychosocial problems ranged from 26 – 41 % in those who reported no sexual violence and without any children, and 34–61 % for those who reported sexual violence but without any children, and 67–76 % for those who reported sexual violence and have children (Fig. 1).

#### Categories of sexual violence and dimensions of psychosocial outcomes

The odds of reporting adverse psychosocial problems were increasingly higher in those with a history of sexual violence but with no children and highest for those who reported sexual violence with children as a consequence compared to those with no history of sexual violence and no children (Table 2). In all the analyses, we adjusted for age at the time of abduction and duration in captivity. Similarly, we compared formerly abducted girls who reported sexual violence without children and those with children. Formerly abducted girls who bore children as a result of sexual violence were at an increased risk of between 47 – 90 % of psychosocial problems compared to those who were sexually abused but did not bear children as a consequence (Table 2).

**Table 2:** Logistic regression analyses: attributable risks of sexual violence on psychosocial outcomes adjusted for age and duration in captivity

Psychosocial outcomes	Participants	No sexual violence, no children	Sexual violence with no children	Sexual violence with children	Sexual violence with no children	Sexual violence with children
		Reference	OR: (95 % CI)	OR: (95 % CI)	Reference	OR: (95 % CI)
Depression/anxiety	210	1.00	2.23 (1.22 – 4.05)	5.37 (1.45–19.90)	1.00	1.53 (1.21–1.85)
Psychotic symptoms	210	1.00	1.38 (0.78–2.47)	1.40 (0.41–3.21)	1.00	1.17 (0.87–1.58)
Conduct problems	210	1.00	1.33 (0.72–2.48)	1.37 (0.37–2.17)	1.00	1.13 (0.82–1.56)
Somatic complaints	210	1.00	2.01 (1.13–3.57)	6.59 (1.80–24.11)	1.00	1.55 (1.14–2.09)
Stigma/discrimination	210	1.00	3.90 (2.14–7.12)	13.85 (3.73–51.42)	1.00	1.90 (1.39–2.61)
Community relations	210	1.00	2.16 (1.21–3.86)	4.37 (1.26–11.10)	1.00	1.49 (1.19–1.79)
Daily functioning	210	1.00	2.00 (1.12–3.56)	4.02 (1.24–13.02)	1.00	1.47 (1.17–1.77)

*OR* Odds Ratio, *CI* Confidence Intervals

## Discussion

### Main findings

These analyses sought to extend previous research by assessing the prevalence and the impact of sexual violence on the odds of reporting adverse psychosocial problems in survivors of sexual violence in Northern Uganda. The results provide a significant contribution and insight into the impact of not only sexual violence but the burden of caring for children born as a result of sexual violence on psychosocial outcomes in survivors. Many notable findings emerged from our analyses. First, up to 65 % of formerly abducted girls reported experiencing sexual violence while in rebel captivity. Second, the risk of psychosocial problems increases with abduction only, abduction with sexual violence only, and abduction with sexual violence and bearing children as a consequence. However, the effect of sexual violence is still strong even on those who are sexually violated but without children. The impact of different levels of sexual violence was not equally manifest on all aspects of psychosocial outcomes in the study. Different categories of survivors of sexual violence experienced different levels of psychosocial outcomes except psychotic symptoms and conduct problems. These findings suggest that exposure to different levels of sexual violence may not have the same effects on various dimensions of psychosocial outcomes. Prior to considering these findings further, a number of methodological limitations need to be considered.

### Limitations

The findings of our study need to be interpreted with caution due to a number of limitations. First, although the sample was generally representative of the population of formerly abducted girls on core population characteristics, we cannot rule out the possibility of selection bias arising from those with a history of sexual abuse before or after abduction. Second, the cross-sectional design of this study makes it impossible to determine whether the effects of sexual violence regard the incidence of psychosocial problems, their duration, or both. In addition, data on both exposure and outcomes came from the same participants. Third, the sources of information on psychosocial problems in the current study were behaviour checklists. Consequently, the results are only indicative of the possible magnitude of psychosocial problems perceived by survivors of sexual violence. Fourth, shame and cultural barriers might have limited gathering of accurate data. Sex and sexual violence are taboo subjects in the culture of the participants. Fifth, it is not possible to compare participants in this study with other groups because the base rates of psychosocial outcomes and difficulty in disclosure are unknown. In addition, we did not have a control group. Last, male victims of sexual abuse were not included in this study due to the small number that reported sexual abuse. This may be due to cultural pressures that make male victims less likely to disclose sexual violence meted against them making them deal with the adverse psychological outcomes on their own. Previous studies show that this can lead to worse outcomes such as delinquency, crime, or perpetrating sexual violence [23]. More research is needed to examine other contexts in which sexual violence occur outside war, considering the occurrence of, and confluence of different factors and forms of vulnerabilities such as poverty and lack of social support.

### Strengths

In spite of these methodological limitations, our study has a number of strengths too. First, to the best of our knowledge, our study is first one to assess the impact of sexual violence on multiple dimensions of psychosocial outcomes concurrently in a single cohort of survivors of sexual violence in a low income setting. Psychosocial problems limit post-war adjustment and may lead to further mental health problems; failure to access services limits opportunities and reduces quality of life of survivors of sexual violence. Second, we used data from a relatively large cohort using a robust, locally developed, and validated measure of psychosocial problems and sexual violence directly obtained from the survivors [19, 20]. Third, we assessed psychosocial problems more than six years after the war ended. Our results were therefore not contaminated by any on-going war, thus increasing the reliability of the findings.

### Implications

The findings in the current study provide initial evidence that the impact of sexual violence is different in various categories of survivors. Previous studies regarded survivors of sexual violence as a homogenous group [10, 11, 13, 15] without considering the possibility that that there could be different categories of survivors of sexual violence such as those with or without children born as a result of their sexual ordeal. In addition, our findings highlight the additional psychosocial burden of care for children born as a consequence of sexual violence, being single parents, the stigma associated with being a formerly abducted girl, and being sexually violated, all of which have not been recognised in previous studies. Except for psychotic symptoms and conduct disorder, we have been able to demonstrate that bearing children as a consequence of sexual violence indeed has adverse psychosocial consequences for formerly abducted girls. Last, by dichotomising the psychosocial outcomes prior to the analyses, although arbitrarily, allows our study greater clinical and public health relevance.

Previous studies with war-affected populations in Sierra Leone [10], the Democratic Republic of Congo [11], in Liberia [13], and in Northern Uganda [15], all found evidence that sexual violence was associated with adverse psychosocial outcomes in survivors. However, in all these studies, the formerly abducted girls were considered as a homogenous group. Our findings are not only in line with and are a useful addition to these evidence but go further to suggest that the impact of sexual violence on psychosocial outcomes depends, to a large degree, on having children born as a consequence of sexual violence. This is consistent with the hypothesis that that the risks of adverse psychosocial outcomes in formerly abducted girls and young women increases with sexual violence and bearing children as a consequence. Our study is therefore the first to categorise survivors of sexual violence with the view of trying to gain insight into the impact of sexual violence on formerly abducted girls.

The processes between the experiences of sexual violence to psychosocial outcomes have been discussed in previous studies [10, 11, 13, 15, 24]. Specifically, post-war environmental inequities related to survivors of sexual violence, especially those with children such as access to health care [15], education [24], and the burden of child care may affect survivors’ well-being. In addition, poverty abounds in villages where survivors of sexual violence have been reintegrated [24]. Moreover, negative traditional views about having children outside wedlock and as a result of sexual violence is a source of chronic stress that impacts on the relationship between survivors and the community in which they have been integrated [6]. It is also possible that, although sexual violence has adverse psychosocial consequences, personal vulnerabilities such as personality problems, social skills, mastery, poverty, and coping skills may be associated with increased risk to psychosocial well-being. It is also possible that being a single parent, the stigma associated with that status, and the additional practical and economic burdens that the situation incurs are associated with adverse psychosocial situation.

The adverse effects of sexual violence on psychosocial well-being such as depression, somatic complaints, stigma, among others, may provide the context for a confluence of other stressful life events to occur and may signal the beginning of a negative spiral into further psychosocial problems. Consequently, survivors of sexual violence are likely to develop more mental health problems as a consequence, have reduced opportunities in life, and live in an environment of long-term and systematic stressors and circumstances [25]. These, coupled with relationship problems, poor personal resources, poor coping strategies, and limited educational and job opportunities, may all lead to entrenched a vicious cycle of poverty and psychosocial problems [26].

### Next steps

To the extent that the undesirable effects of sexual violence on psychosocial well-being may provide the context for a confluence of other risk factors, it is critical to move on from quantifying the odds of psychosocial outcomes for sexual violence to examine the impacts of multiple exposures of these risk factors, how they interact, and the mechanisms through which they impact survivors. Currently, we are constrained by limited data on these other risk factors and difficulties of delineating cause and effect. In spite of these constraints, the results of our study support the hypothesis that vulnerability for adverse psychosocial outcomes in survivors of sexual violence depends on exposure and whether the exposure results into a child or children. This may have implications for research, policy, and clinical practice for sub-populations that may be at risk and in need of interventions to promote psychosocial wellbeing.

Research efforts should be directed at devising strategies to mitigate psychosocial problems such as symptoms of depression/anxiety, somatic complaints, poor functioning, and perceptions of stigma and poor community relation for survivors of sexual violence from a public health point of view. For example, group interpersonal psychotherapy for depression has been shown to be efficacious in reducing depression and dysfunction in rural settings in Uganda [27]. In addition, further research is required to develop and evaluate interventions to change attitudes towards survivors of sexual violence and address myths surrounding sexual violence and its consequences. Although reducing psychosocial problems is crucial, it is imperative to address post-war environmental stressors which have been demonstrated to reduce negative psychosocial outcomes [16]. Future studies should also consider the role of factors such as social support, mentoring from community members, coping skills, and self-efficacy in reducing psychosocial problems. Similarly, female survivors of protracted violent wars in less resourced settings should also be a focus of future research unlike previous studies that neglected this group [27]. For policy makers, the implication of this study is to direct interventions to formerly abducted girls who were victims of sexual violence. For example, programmes to improve personal resources to provide for child care or empowering families to care for the children while survivors of sexual violence return to school to increase their opportunities for employment may be considered. Policies that take into consideration negative cultural practices and are sensitive to gender differences to enable survivors of sexual violence be prioritised in post-war development agenda would be particularly beneficial. Clinicians and other health workers involved in primary care should be aware of the sub-population differences among survivors of sexual violence and recognise psychosocial problems and their impact on survivors of sexual violence, especially those with children as a consequence.

## Conclusion

This study shows that sexual violence has adverse psychosocial effects on survivors with the adverse effects stronger for those who were abducted and sexually abused but did not have children and strongest for those who were sexually abused and had children as a consequence. Although sexual violence may contribute to the risk of psychosocial problems in formerly abducted girls differently and varies with different levels or consequences of sexual violence experienced while in captivity, emphasis should be on sexual violence in general. Policies and interventions to reduce psychosocial problems may require long-term interventions on sexual violence, stigma associated with abduction, single parenthood, and sexual violence as important risk factors for psychosocial problems.
